# Hypomyelination and Oligodendroglial Alterations in a Mouse Model of Autism Spectrum Disorder

**DOI:** 10.3389/fncel.2018.00517

**Published:** 2019-01-11

**Authors:** Mariana Graciarena, Araceli Seiffe, Brahim Nait-Oumesmar, Amaicha M. Depino

**Affiliations:** ^1^Brain and Spine Institute, Inserm U1127, Sorbonne Universités/Université Pierre & Marie Curie UMRS 1127, CNRS UMR 7225, Paris, France; ^2^Departamento de Fisiología, Biología Molecular y Celular, Facultad de Ciencias Exactas y Naturales, Universidad de Buenos Aires, Buenos Aires, Argentina; ^3^Instituto de Fisiología, Biología Molecular y Neurociencias (IFIBYNE), CONICET-Universidad de Buenos Aires, Buenos Aires, Argentina

**Keywords:** autism spectrum disorder, myelin, oligodendrocytes, valproic acid, mouse

## Abstract

Autism spectrum disorders (ASDs) are neuropsychiatric diseases characterized by impaired social interaction, communication deficits, and repetitive and stereotyped behaviors. ASD etiology is unknown, and both genetic and environmental causes have been proposed. Different brain structures are believed to play a role in ASD-related behaviors, including medial prefrontal cortex (mPFC), hippocampus, piriform cortex (Pir), basolateral amygdala (BLA) and Cerebellum. Compelling evidence suggests a link between white matter modifications and ASD symptoms in patients. Besides, an hypomyelination of the mPFC has been associated in rodents to social behavior impairment, one of the main symptoms of ASD. However, a comparative analysis of myelination as well as oligodendroglial (OL)-lineage cells in brain regions associated to social behaviors in animal models of ASD has not been performed so far. Here, we investigated whether OL-lineage cells and myelination are altered in a murine model of ASD induced by the prenatal exposure to valproic acid (VPA). We showed an hypomyelination in the BLA and Pir of adult VPA-exposed mice. These results were accompanied by a decrease in the number of OL-lineage cells and of mature OLs in the Pir, in addition to the mPFC, where myelination presented no alterations. In these regions the number of oligodendrocyte progenitors (OPCs) remained unaltered. Likewise, activation of histone deacetylases (HDACs) on OL-lineage cells in adulthood showed no differences. Overall, our results reveal OL-lineage cell alterations and hypomyelination as neuropathological hallmarks of ASD that have been overlooked so far.

## Introduction

Autism spectrum disorders (ASDs) are a group of neurodevelopmental disorders characterized by impaired social interaction, communication deficits, and repetitive, stereotyped behaviors (American Psychiatric Association, 2013). These symptoms appear early in life and persist during adulthood. ASD etiology is unknown, and both genetic and environmental factors can contribute to its development (Betancur, 2011). There are currently no treatments that can treat ASD symptoms altogether.

There is no current consensus about the underlying neuropathology in ASD, and different brain structures have been proposed to play a role in ASD symptoms. In humans, the medial prefrontal cortex (mPFC) was identified as one of the main brain areas whose connectivity is affected in ASD subjects (Cheng et al., 2015), while other areas also exhibit altered neuronal number or volume in people diagnosed with ASD, e.g., the basolateral amygdala (BLA; Lin et al., 2013; Wegiel et al., 2014), the Hippocampus (Sussman et al., 2015) and the cerebellum (Fatemi et al., 2012; Sussman et al., 2015).

Different animal models for ASD have been validated for studying the cellular and molecular alterations that lead to altered behavior in these disorders. In particular, prenatal exposure to valproic acid (VPA) results in reduced sociability and increased repetitive behaviors (Lucchina and Depino, 2014; Campolongo et al., 2018). In addition, other cellular and molecular alterations related to ASD are also observed in animals prenatally exposed to VPA, e.g., neuroinflammation (Lucchina and Depino, 2014), and altered excitation/inhibition balance (Cunningham et al., 2003). Finally, this animal model also shows alterations in neuronal function in a region recently involved in social behaviors, the Piriform cortex (Pir; Choe et al., 2015; Campolongo et al., 2018).

Increasing evidence links neuropsychiatric conditions with white matter alterations (Fields, 2008). Concerning ASD, white matter integrity and myelin thickness was found altered in the brains of ASD patients, particularly in the inter-hemispheric circuitry and in the corpus callosum (Travers et al., 2012; Ameis et al., 2016). Furthermore, a link between myelination of the mPFC and social exploration deficits has been described in mice (Liu et al., 2012; Makinodan et al., 2012).

Myelin, a multilamellar structure that ensheathes axons and allows for fast saltatory conduction of action potentials, is produced by oligodendrocytes (OLs) both during development and in adult life in vertebrates. Myelin has been recently shown to respond to and participate in the activity and fine-tuning of neuronal networks, which results in the modulation of speed and synchronicity of action potentials as well as provides metabolic support to axons (Pajevic et al., 2014; Filley and Fields, 2016). Moreover, oligodendrocyte progenitor cells (OPCs) are synaptically innervated by neuronal fibers throughout the central nervous system (Bergles et al., 2000; Fröhlich et al., 2011) thus constituting a potential mechanism for this bilateral communication.

Interestingly, *de novo* myelination continues throughout life (Bergles and Richardson, 2015), and it can contribute to adult neural plasticity, as the conduction properties of myelinated neuronal circuits may undergo important transformations. In this sense, myelin may play a key role in the neuronal circuit dysfunctions that occur in neuropsychiatric diseases such as ASD. However, a comparative analysis of the myelination status and OL differentiation in brain regions functionally related to ASD symptoms has not been attempted to date.

Here, we investigated whether myelination and OL-lineage cells were maintained in VPA-treated mice, a well-characterized ASD murine model that recapitulates the main aspects of this condition. Our data show long-term alterations in myelin content and in myelin-producing cells in the mPFC, BLA and Pir, three brain regions functionally associated with social behavior. These changes were not associated to H3 acetylation, an indicator of active DNA transcription, in adult OL-lineage population. Overall, these results constitute a fundamental characterization of myelin integrity in an ASD model, suggesting the potential contribution of a myelination deficit in this psychiatric condition.

## Materials and Methods

### Animals

Outbred CrlFcen:CF1 mice were obtained from the animal house at the Faculty of Exact and Natural Sciences (FCEN), University of Buenos Aires (UBA, Argentina). Animals were housed on a 12:12 light:dark cycle and 18–22°C temperature, with food and water *ad libitum*. All animal procedures were performed according to the regulations for the use of laboratory animals of the National Institute of Health, Washington DC, USA, and approved by the Institutional Commission for Care and Use of Laboratory Animals (CICUAL Protocol No. 6/2, FCEN, UBA, Argentina). Eight-to-ten week old male mice were mated with nulliparous age matching female mice. Female mice were controlled daily for presence of vaginal plugs, and whenever present this day was considered the embryonic day (E) 0.5.

### VPA Prenatal Treatment

On E12.5, pregnant mice were subcutaneously injected with 600 mg/kg of VPA sodium salt (Sigma, St. Louis, MO, USA) resuspended in saline solution or saline solution alone, and housed individually. The parturition day was registered as postnatal day 0 (PD0), and the cage bedding was not changed during the first postnatal week to avoid nest and maternal care alterations. We studied five male offspring animals per group for immunofluorescence and 3–4 male animals per group for electron microscopy (EM), obtained in two independent cohorts.

### Social Interaction Test

At PD60, animals were evaluated in the social interaction test as previously described (Depino et al., 2011; Lucchina and Depino, 2014; Campolongo et al., 2018). Briefly, mice were habituated for 10 min to a 40 × 15 cm black rectangular arena divided in three interconnected chambers placed under dim light (10 lx). A clear Plexiglass cylinder (7.5 cm of diameter, with several holes to allow for auditory, visual, and olfactory investigation) was placed in each side compartment at the beginning of the test. Prior to the start of each test, one of the end chambers was randomly designated as the “non-social side” and the other as the “social side.” Animals were placed in the central compartment and allowed to explore for 10 min (habituation). Then, an unfamiliar, young (3 weeks) CF1 male mouse (social stimulus) was placed in one of the cylinders (social side), and an object (plastic 3 cm-tall cylinder) was placed in the other cylinder (non-social side). Social interaction was evaluated during a 10 min period. The time the subject spent sniffing the social stimulus or the non-social stimulus (with the nose <1 cm distance to a hole of the cylinder) was recorded manually using the ANY-maze video-tracking system (Stoelting, IL, USA) by an experimenter blind to treatments. The entire apparatus was cleaned with a 20% ethanol solution between tests to eliminate odors.

The preference for the social stimulus was calculated as the difference between the time spent in the social side vs. the time spent in the non-social side.

### Tissue Processing

At PD90, mice were deeply anesthetized with 80 mg/kg ketamine chlorhydrate and 8 mg/kg xylazine. Next, they were transcardially perfused with heparinized saline followed by cold 4% paraformaldehyde in 0.1 M phosphate buffer (PB) of pH 7.2. Brains were removed and postfixed in PFA at 4°C, then cryopreserved in a 30% sucrose solution in PB at 4°C until full immersion was observed. Subsequently, brains were frozen with isopentane and 30 μm coronal sections were obtained using a cryostat (Leica Biosystems, Nussloch, Germany), which were either used immediately for immunostaining or stored at −20°C in a cryopreservative solution.

### Immunostaining

Free-floating immunostaining was performed as follows: briefly, every sixth coronal section was incubated in PB for 15 min. Sections were then incubated in blocking solution, 4% bovine serum albumin (BSA) in PB with 0.1% Triton X-100 for 45 min. Primary antibodies were diluted in the blocking solution and sections were incubated overnight at 4°C. On the following day sections were washed three times with 0.1 M PB and incubated in secondary antibodies diluted in 0.1 M PB for 2 h in the dark. Sections were then washed with 0.1 M PB and mounted in Fluoromount-G (Thermo Fisher Scientific, Waltham, MA, USA).

The primary antibodies used were anti-MBP; anti-myelin basic protein (AB980, Millipore, MA, USA), anti-Olig2 (AB9610, Millipore), anti-NG2 (AB5320, Millipore), anti-CC1 (OP80, Millipore), and anti-acetylated histone-3 (anti-AcH3; Santa Cruz Biotechnology, Dallas, TX, USA). Secondary antibodies used were Alexa 488 and Alexa 568-conjugated donkey anti-rat, mouse or rabbit (Jackson Laboratories, West Grove, PA, USA).

For each brain structure of interest, 20–25 z-stack images were taken 1 μm apart, spanning the entire depth of the tissue section (30 μm) using a Confocal Olympus FV1200 microscope with 20× (numerical aperture-NA 0.40) and 40× (NA 0.65) objectives for BLA/Pir and mPFC respectively. Images with a resolution of 0.31 (mPFC) and 0.62 microns (BLA and Pir) were analyzed on maximal projection with NIH ImageJ^1^ using a macro that allows for systematically subtracting the same background in all images and quantifying the fluorescence above a threshold in the region of interest.

### Electron Microscopy

For ultrastructural visualization of myelinated axons in the PFC, BLA and Pir, PD90 mice were perfused with 1% glutaraldehyde and 4% paraformaldehyde in PB. Brains were immersed in the same fixative for 1 h. After rinsing brains with PB, 100-μm sagittal sections spanning the area of interest were obtained with a vibratome (Leica VT1000S). In order to standardize the quantifications and due to potential differences in myelination within each region along the lateral axis, we chose the exact same sagittal section of each region in all brains to proceed with. We subsequently incubated them in 2% osmium tetroxide (OsO_4_) for 1 h, rinsed them in distilled water and contrasted them with uranyl acetate 5% for 30 min. Dehydration was achieved by a graded series of ethanol and clearing in acetone. Sections were then embedded in epoxy resin (Agar Scientific, UK) and incubated at 56°C for 48 h to allow for polymerization. Polymerized Epon blocks were observed at the dissection microscope and the area of interest was manually microdissected. Next, dissected blocks were cut in 1 μm semi-thin sections with an ultramicrotome (UC7, Leica) which were stained with toluidine blue and visualized until the entire tissue area was observed. Then, serial ultra-thin sections (~70 nm thick) were collected onto copper grids (about 3–4 sections per grid) and visualized at the transmission electronic microscope (Hitachi HT7700). Electron micrographs were taken at ×18,000 magnifications using an integrated AMT XR41-B camera (2,048 × 2,048 pixels).

Measurements were made using NIH ImageJ. G-ratio was calculated by dividing the axonal diameter by the total diameter of the axon including the surrounding myelin sheath. Both diameters were calculated by averaging two perpendicular measurements. For each condition, the g-ratio of at least 250 randomly selected axons was calculated.

### Statistical Analysis

Sample data were tested for normal distribution. Unpaired and paired Student’s *t*-test were used for comparisons. Correlations were performed and analyzed using the Graphpad Prism software, where Pearson’s coefficient, *R* square and *p* values were calculated. In all cases, statistical significance was considered when *p* < 0.05.

## Results

### Prenatal VPA Exposure Affects Myelination in Brain Regions Linked to Social Behavior

**Figure 1:**
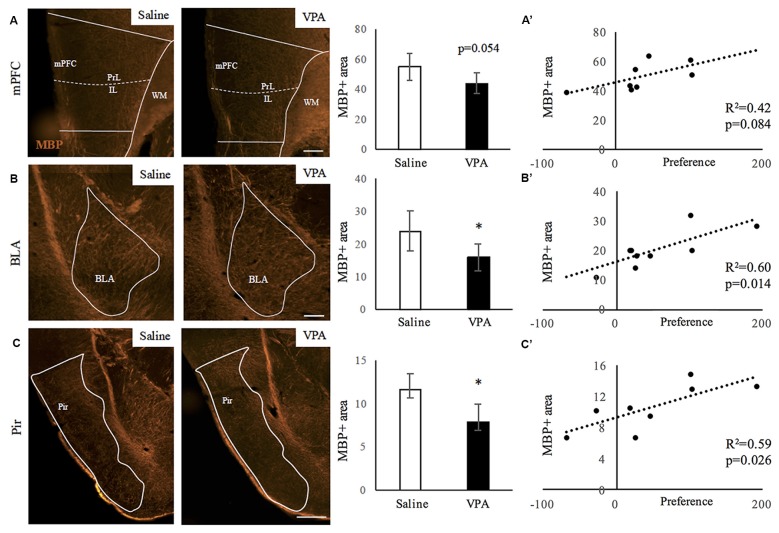
Myelin content decreases upon valproic acid (VPA) treatment in brain areas linked to social behavior. **(A–C)** Myelin basic protein (MBP) immunostaining in saline- and VPA-treated adult mouse brains (representative images) and estimated percentage of positive surface in the following areas: **(A)** medial prefrontal cortex (mPFC; mean: saline: 55.01 ± 9.12; VPA: 44.17 ± 7.08; Student’s *t*-test: *t* = 1.878, *p* = 0.054). **(B)** Basolateral amygdala (BLA; mean: saline: 23.89 ± 6.05; VPA: 16.01 ± 4.11; Student’s *t*-test: *t* = 2.211, *p* = 0.031). **(C)** Piriform cortex (Pir; mean: saline: 11.60 ± 1.81; VPA: 7.92 ± 1.97; Student’s *t*-test: *t* = 2.845, *p* = 0.015). *p < 0.05 (Student’s *t*-test). Scale bars: 100 μm. *N* = 4–5 mice/treatment. **(A’–C’)** Correlation between preference for social stimulus and MBP positive area in mPFC **(A’)**, BLA **(B’)** and Pir **(C’)**. Significance: *p* < 0.05 (Pearson correlation analysis).

Mice prenatally exposed to VPA showed reduced sociability in adulthood (Supplementary Figure S1). We then aimed to study whether VPA treated mice presented alterations in myelin content in areas related to social behavior. As a first approach, we performed immunostainings for MBP detection—an essential protein component of myelin—and quantified the relative MBP-positive area in the following regions: mPFC, BLA, Pir, hippocampus and cerebellum. Figure 1 shows the comparisons between mice prenatally exposed to VPA or saline (control). Whereas significant differences were observed in the BLA and Pir (Figures 1B,C) as well as a tendency in the mPFC (Figure 1A), the hippocampus and lobule VII of the cerebellum presented an equivalent amount of myelin in both experimental groups (Supplementary Figures S2A,B). We performed correlations between the preference for the social stimulus and the MBP positive area in all animals (Figures 1A’–C’). Whereas in the mPFC these parameters do not correlate (*r* = 0.64; *R*^2^ = 0.42; *p* = 0.084), they do show a significant positive correlation in both the BLA (*r* = 0.78; *R*^2^ = 0.60; *p* = 0.014) and the Pir (*r* = 0.77; Pir: *R*^2^ = 0.59; *p* = 0.026). These results suggest a link between the VPA-triggered social deficits and the hypomyelination observed.

**Figure 2:**
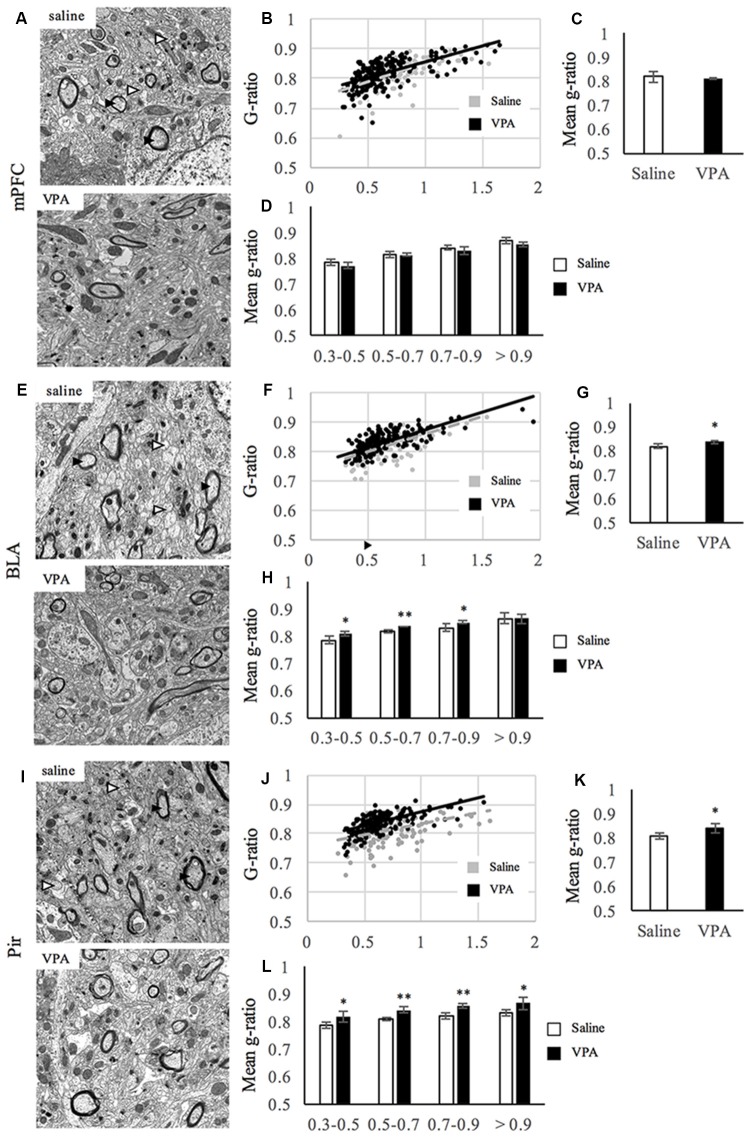
Myelin thickness is impaired upon VPA treatment in brain areas linked to social behavior. **(A,E,I)** Representative electron micrographs of transversal sections from mPFC **(A)**, BLA **(E)**, Pir **(I)** of saline- and VPA treated animals. Arrowheads in the pictures of saline-treated groups show examples of myelinated (black) and unmyelinated (white) axons. **(B,F,J)** Dot plot of the G-ratio distribution along axonal diameters for saline- (gray) and VPA- (black) treated animals at the mPFC **(B)**, BLA **(F)** and Pir **(J)**. **(C,G,K)** Mean G-ratio at the mPFC (**C**; mean G-ratio: saline: 0.819 ± 0.012; VPA: 0.813 ± 0.001; Student’s *t*-test: *t* = 0.497, *p* = 0.645), BLA (**G**; mean G-ratio: saline: 0.819 ± 0.004; VPA: 0.837 ± 0.004; Student’s *t*-test: *t* = 2.81, *p* = 0.0375) and Pir (**K**; mean G-ratio: saline: 0.809 ± 0.003; VPA: 0.840 ± 0.0105; Student’s *t*-test: *t* = 2.8, *p* = 0.049). **(D,H,L)** G-ratio values along ranges of axonal diameters at the mPFC [**D**; means: (0.3–0.5) saline: 0.787 ±0.012; VPA: 0.773 ± 0.011; Student’s *t*-test: *t* = 1.46, *p* = 0.109; (0.5–0.7) saline: 0.815 ± 0.011; VPA: 0.814 ±0.005; Student’s *t*-test: *t* = 0.234, *p* = 0.413 (0.7–0.9) saline: 0.843 ± 0.008; VPA: 0.830 ± 0.013; Student’s *t*-test: *t* = 1.38, *p* = 0.0119 (>0.9) saline: 0.870 ± 0.013; VPA: 0.857 ± 0.008; Student’s *t*-test: *t* = 1.40, *p* = 0.117], BLA [**H** (0.3–0.5) saline: 0.787 ± 0.016; VPA: 0.809 ± 0.006; Student’s *t*-test: *t* = −2.15, *p* = 0.042 (0.5–0.7) saline: 0.819 ± 0.006; VPA: 0.836 ± 0.001; Student’s *t*-test: *t* = −4.27, *p* = 0.004 (0.7–0.9) saline: 0.832 ±0.013; VPA: 0.851 ± 0.005; Student’s *t*-test: *t* = −2.25, *p* = 0.037 (>0.9) saline: 0.865 ± 0.020; VPA: 0.865 ± 0.018; Student’s *t*-test: *t* < 0.001, *p* = 0.499] and Pir [**L** (0.3–0.5) saline: 0.786 ± 0.010; VPA: 0.820 ± 0.019; Student’s *t*-test: *t* = −2.73, *p* = 0.026 (0.5–0.7) saline: 0.809 ± 0.006; VPA: 0.841 ± 0.011; Student’s *t*-test: *t* = −4.18, *p* = 0.007 (0.7–0.9) saline: 0.820 ± 0.009; VPA: 0.854 ± 0.008; Student’s *t*-test: *t* = −4.77, *p* = 0.004 (>0.9) saline: 0.830 ± 0.011; VPA: 0.867 ± 0.022; Student’s *t*-test: *t* = −2.47, *p* = 0.034], **p < 0.01, *p < 0.05 (Student’s *t*-test). *N* = 3 mice per condition.

Next, we extended our analyses of the observed hypomyelination and performed EM in an independent cohort of animals. For that, we studied at the EM level the areas where a different amount of myelin content was observed, the mPFC, the BLA and the Pir, where we quantified the G-ratio as a measure of myelin sheath thickness (Figures 2A–L). In the mPFC, we found no differences in the G-ratio distribution along axonal diameters (Figures 2A,B) or the mean G-ratio (Figures 2A,C). Moreover, the G-ratio was not affected in any population harboring a specific range of axonal diameters (Figure 2D). Consistently, the distribution of axonal diameters was not affected by VPA treatment (Supplementary Figure S3A). In the BLA, prenatal VPA treatment affected the mean G-ratio (Figures 2E,G). Here, the slope of the curve was slightly shifted upwards in the VPA group where axonal diameters are smaller (Figure 2F). In the same line, and while axonal diameter distribution was preserved (Supplementary Figure S3B), the G-ratio was affected in axons of diameters ranging from 0.3 μm to 0.9 μm but not over 0.9 μm (Figure 2H), indicating that the effect of VPA treatment in the BLA is extensive but exclusively on fibers with axonal diameters under 0.9 μm. In the Pir, the mean G-ratio was affected upon prenatal VPA treatment (Figures 2I,K) and the distribution curve showed a shift upwards in the VPA group (Figure 2J). Consistently, the G-ratio was affected in axons of all sizes (Figure 2L), whereas the axonal diameters themselves showed no differences in their distribution upon VPA treatment (Supplementary Figure S3C). Altogether, our data indicate that myelination, and G-ratio in particular, is affected in both BLA and Pir regions of VPA-treated animals.

### Prenatal Exposure to VPA Reduces OL-Lineage Numbers in the mPFC and Pir, but Not in the BLA

**Figure 3:**
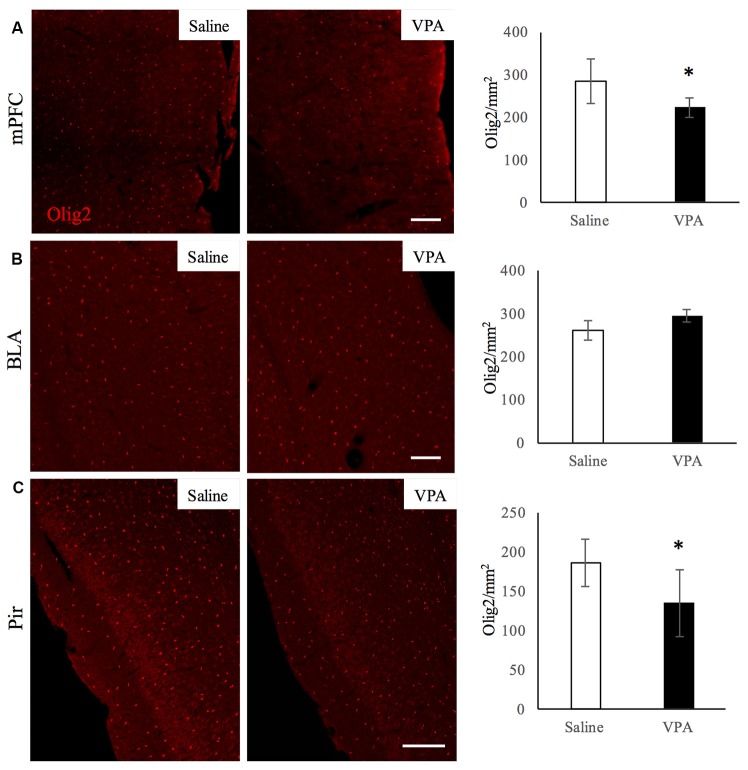
VPA treatment reduces oligodendroglial (OL)-lineage cell numbers in brain areas linked to social behavior. Olig2 immunostaining in saline- and VPA-treated adult mouse brains (representative images, left panels) and Olig2-positive cell quantifications (right panels) in the following areas: **(A)** mPFC (mean: saline: 284.43 ± 53.29; VPA: 222.29 ± 23.80; Student’s *t*-test: *t* = 2.12, *p* = 0.039); **(B)** BLA (mean: saline: 260.63 ± 23.58; VPA: 285.50 ± 17.62; Student’s *t*-test: *t* = −1.83, *p* = 0.052); **(C)** Pir (mean: saline: 186.69 ± 30.01; VPA: 135.08 ± 42.91; Student’s *t*-test: *t* = 2.13, *p* = 0.035). *p < 0.05, Student’s *t*-test. Scale bars: 100 μm. *N* = 4–5 mice/treatment.

Next, we investigated whether the impaired myelination was accompanied with a decrease in OL-lineage cells. For that, we quantified the number of Olig2-positive cells in the aforementioned regions (Figure 3) of saline- and VPA-treated mice. We observed that the number of Olig2-positive cells was lower in two regions of VPA-treated mice: the mPFC (Figure 3A) that shows equivalent levels of myelination than saline-treated group; and the Pir which has a reduced myelin content (Figure 3C). In contrast, in the BLA, where myelination was also reduced, the OL-lineage cell numbers were similar in both groups (Figure 3B). These results suggest that different mechanisms can underlie the lower myelination observed in the Pir and BLA. In the other regions where myelination was unaltered upon VPA treatment, the hippocampus and cerebellum, OL-lineage cell numbers were equivalent in both experimental groups (Supplementary Figures S4A,B). Hence, a decrease in myelination can be associated with less oligodendroglial cells as it occurs in the Pir, although not exclusively, as observed in the BLA.

### Prenatal VPA Exposure Reduces Mature OL Numbers in the mPFC and Pir, but Not in the BLA

**Figure 4:**
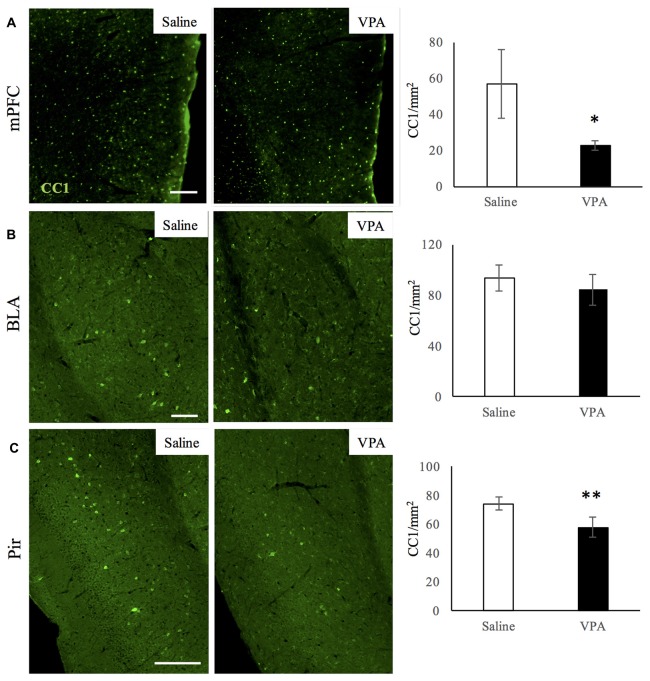
VPA treatment reduces mature OL numbers in brain areas linked to social behavior. CC1 immunostaining in saline- and VPA-treated adult mouse brains (representative images, left panels) and CC1 positive cell quantifications (right panels) in the following areas: **(A)** mPFC (mean: saline: 53.93 ± 19.22; VPA: 22.77 ± 2, 69; Student’s *t*-test: *t* = 3.52, *p* = 0.006); **(B)** BLA (mean: saline: 93.79 ± 10.21; VPA: 84.42 ± 12.32; Student’s *t*-test: *t* = 1.17, *p* = 0.143); **(C)** Pir (mean: saline: 74.31 ± 4.46; VPA: 57.99 ± 6.89; Student’s *t*-test: *t* = 3.53, *p* = 0.008). *p < 0.05, **p < 0.01, Student’s *t*-test. Scale bars: 100 μm. *N* = 4–5 mice/treatment.

We next studied whether a specific stage of the OL-lineage cells was differentially affected in VPA-treated animals. In order to answer this, we performed immunostainings for CC1 and for NG2 markers, to identify mature OLs and OPCs respectively. NG2 cells did not display any difference in number in both the mPFC and Pir (Supplementary Figures S5A–F), indicating that OPC numbers in adulthood are not affected by VPA treatment. However, the number of mature OLs did decrease in both of these regions (Figures 4A,C), whereas in the BLA the mature OLs were not affected (Figure 4B), showing a distribution similar to what we observed for the whole OL-lineage population.

### Histone Acetylation Is Not Affected in Oligodendroglial Cells in Any of the Regions Studied

**Figure 5:**
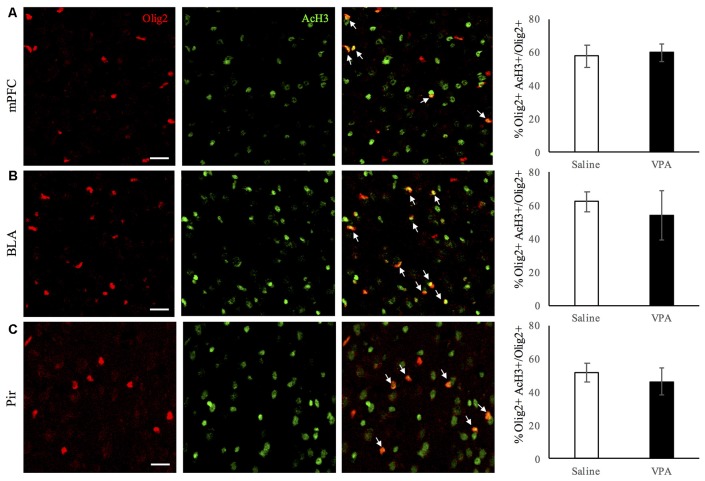
Histone acetylation in OL-lineage cells is unaltered upon VPA-treatment in brain areas linked to social behavior. Double Olig2/acetylated histone-3 (AcH3) immunostaining in saline- and VPA-treated adult mouse brains (representative images, left panels), and estimated percentage of acetylated histone-3 (AcH3) positive cells among Olig2 positive cells (right panels) in the following areas: **(A)** mPFC (mean: saline: 57.55 ± 6.76; VPA: 59.78 ± 5.23; Student’s *t*-test: *t* = −0.49, *p* = 0.321); **(B)** BLA (mean: saline: 62.40 ± 6.03; VPA: 54.29 ± 14.83; Student’s *t*-test: *t* = 1.073, *p* = 0.162); **(C)** Pir (mean: saline: 51.71 ± 5.43; VPA: 46.31 ± 7.87; Student’s *t*-test: *t* = −1.038, *p* = 0.173). *p < 0.05, Student’s *t*-test. Scale bars: 100 μm. *N* = 4–5 mice/treatment.

Last, as it has been shown that histone deacetylation is required for oligodendrocyte differentiation (Shen et al., 2005, 2008; Ye et al., 2009; Liu et al., 2012), we explored the possibility that the decrease in myelination was related to persistent epigenetic changes in OL-lineage cells in VPA-treated mice. We assessed the proportion of Olig2-positive OL-lineage cells that expressed AcH3, a marker of histone deacetylase recruitment to promoter regions. As it can be observed in Figures 5A–C, this proportion remains unchanged in the regions analyzed, therefore suggesting that persistent histone deacetylation might not participate in neither the reduction of myelination observed in the BLA and Pir, nor in the changes in OL-lineage cells and mature OLs observed in the Pir and mPFC.

## Discussion

This work shows that in adult mice prenatally exposed to VPA, which display the core symptoms of ASD, the myelin content in some of the main brain regions linked to social behavior (BLA and Pir) is diminished. Moreover, the deficit in myelination in the BLA and Pir regions correlates with levels of sociability observed in these mice, with VPA treated animals showing less sociability and having less MBP levels. EM studies confirm these myelin alterations by showing a thinner myelin sheath (measured as G-ratio) in axons of the BLA and Pir regions in VPA-treated mice. This impairment can be accompanied by a reduction in OL-lineage cells and of mature OLs, or can occur without any perturbation in the number of myelin-producing cells, found in the Pir and BLA regions respectively. Similarly, the OL-lineage cell and mature OL reduction can be reduced in response to VPA treatment without a concomitant decrease in myelin, as it is observed in the mPFC. In addition, in the hippocampus and cerebellum no differences were observed in myelin content nor in OL-lineage cell number upon VPA-treatment. This suggests that in the VPA-treated group, either closely related or different mechanisms may participate in myelination impairment and OL-lineage cell reduction depending on the region analyzed. Finally, histone acetylation in OL-lineage cells is not affected by VPA treatment, therefore it does not account for the observed effects in myelination and/or OL-lineage cell reduction.

A lower myelination has been described in some of the regions studied here in different mouse models that recapitulate ASD-core symptoms. Particularly, in a mouse model of fragile X syndrome, the cerebellum displays a transient reduction in MBP and 2’,3’-cyclic nucleotide 3’-phosphodiesterase (CNP) expression as well as myelination at PD7, later showing complete recovery at PD15 (Pacey et al., 2013). In addition, myelination did also show alterations in the cerebellum of Slc25a12-knockout mice at PD35 (Sakurai et al., 2010). As these studies analyze myelination during development, we cannot rule out a potential transient decrease of myelin in this region at younger stages in VPA-treated animals. Concerning the hippocampus, a decrease in MBP expression has been reported in VPA-exposed rats at PD35, along with fewer OL-lineage cells specifically in CA1 and CA2 areas (Cartocci et al., 2018). We consider that the differences in species and ages analyzed may account for the discrepancies between that study and the results presented here. Lastly, a functional link between myelination of the mPFC and social behaviors have been shown (Sirevaag and Greenough, 1987; Sánchez et al., 1998; Liu et al., 2012; Makinodan et al., 2012).

Similarly, vast evidence shows a link between ASD symptoms and alterations in white matter in ASD patients. For example, decreased myelin thickness in the orbitofrontal cortex—a subregion of the PFC in humans- has been reported in post mortem tissue of ASD patients (Zikopoulos and Barbas, 2010). The fractional anisotropy values (a measure of white matter integrity) were also shown altered in the BLA of ASD patients (Barnea-Goraly et al., 2004), whereas in the Pir the extent of myelination and white matter integrity have not been analyzed in the context of ASD patients, although this region has been associated with social behavior (Richter et al., 2005; Borelli et al., 2009; Campolongo et al., 2018). Overall, these studies reinforce the concept that impairment of myelin development and disruption of white matter tracts in regions implicated in social functioning may contribute to impaired social cognition in ASD.

We observed that in one of the regions analyzed, the Pir, myelin reduction is accompanied with a decrease in mature OL number, whereas OPCs are maintained. This suggests that in this region, reduced myelination may likely result from a failure in the OPC differentiation towards mature OLs. On the contrary, in the BLA, myelin reduction was observed concurrently with an equivalent number of OL-lineage cells and mature OLs. Here, other mechanisms related to the myelination process itself might play a central role in this outcome. It is possible that in this case, defects in the myelination process *per se* were playing a role, regulated by key transducers such as the myelin regulatory factor (MRF), a critical regulator of myelination whose downregulation does not affect differentiation into mature OLs (Emery et al., 2009). Other factors with a similar specific role in myelination are ZFP191 (Howng et al., 2010), Nkx6.2, essential for paranode structure (Southwood et al., 2004), and ErBb3 signaling, which recapitulates the social isolation effects on impaired myelination due to myelinating OL altered morphology (Makinodan et al., 2012). Conversely, we describe that prenatal VPA treatment did not affect myelination in the mPFC, although it caused a reduction in the OL-lineage cells and particularly in the mature-OLs. This phenomenon can be linked to CNP protein function, which is essential for axonal survival but not for myelin assembly. In the absence of this protein, mice developed axonal loss as a consequence of OL dysfunctions; however, the content, ultrastructure and physical properties of myelin are not altered in young mice (Lappe-Siefke et al., 2003). Therefore, as OLs are an essential component for axonal survival, the possibility that OL impaired functions were sufficient for affecting neuronal communication and circuit integrity without altering myelination should also be taken into account.

Another important aspect that should also be considered in order to better understand the differences described here is the fact that OLs are indeed heterogeneous among brain regions in terms of transcriptomic profiles as well as timing of OL maturation and migration (Zhang et al., 2014; Marques et al., 2016). Different subsets of OLs throughout the brain are just beginning to be characterized, thus this information will be valuable to explain different OL involvement in pathological conditions.

Given that the core behaviors of ASD that are recapitulated in the VPA model are generated by neuronal circuit dysfunctions, and that neuronal activity is now known to play a critical role in myelination (Wake et al., 2011; Gibson et al., 2014; Mensch et al., 2015), we cannot rule out the possibility that altered neuronal activity with subsequent social impairment and lower myelination were not just causally related, but that these concomitant modifications were a result of a distorted bidirectional communication. In this sense, the same study that described an impaired myelination in mice that were exposed to a social isolation paradigm, reported that the OL-specific knock out of Neuregulin-ErbB3 signaling, a key pathway for myelination, results in social deficits and defective myelination (Makinodan et al., 2012). Demyelination of the mPFC by cuprizone treatment also resulted in diminished social behavior (Makinodan et al., 2009). Therefore, studies that shed light on the communication between these two processes in the context of VPA treatment, and particularly on whether neuronal activity is involved in the uncoupling of OL axonal support and myelination, would be a valuable step towards a deeper understanding of the reported effects.

We speculated with the hypothesis that epigenetic modifications within the OL-lineage cells might account for the myelination defects in VPA-treated mice. In this sense, it has been shown that in demyelinating conditions, recruitment of histone deacetylases (HDACs) to promoter regions of myelin genes precedes myelin synthesis. As the brain ages, this recruitment becomes more inefficient, thus allowing transcriptional inhibition and in turn preventing myelin gene expression (Liu et al., 2012). The presence of HDACs in the OL cell nuclei is therefore an early indicator of myelin synthesis. VPA is known to have an inhibitory effect on HDACs (Göttlicher et al., 2001). We aimed to determine whether in this model myelination impairment was associated with a difference in the degree of histone acetylation as a consequence of a poor myelin turnover. However, we observed no differences in AcH3 expression in OL-lineage cells between control and VPA groups. This suggests that adult myelin plasticity in response to VPA treatment might not be mediated by changes in nuclear heterochromatin. Nevertheless, we cannot rule out that histone acetylation occurs in earlier stages of development in VPA-treated mice and that they can lead to the reduction in myelin that we observe.

Overall, our results using prenatal VPA treatment establish a fundamental cellular link between two processes that are just beginning to be considered as functionally related. We describe a deficit in myelination in some of the main regions involved in social behavior in an animal model of ASD, which shows key features of this psychiatric disorder. We show that myelin reduction can be unrelated to alterations in the number of OL-lineage cells and of mature OLs, suggesting that depending on the brain region analyzed, different mechanisms mediate this response. While the present approach is robust and non-invasive due to its prenatal characteristics, exploring our hypotheses in different environmental and genetic ASD models would be necessary to confirm the translational implications of our results and to understand additional signatures regarding the correlation between decreased myelination and ASD core symptoms. Thus, our work opens new basic questions that may guide us to a better understanding of the link between OL axonal support, myelination and ASD related behaviors, and that can eventually lead to novel targets for therapeutic interventions in individuals affected by ASD.

## Author Contributions

MG designed and carried out experiments, and wrote the manuscript. AS carried out experiments. AD designed experiments. BN-O and AD co-wrote the manuscript and supervised this study.

## Conflict of Interest Statement

The authors declare that the research was conducted in the absence of any commercial or financial relationships that could be construed as a potential conflict of interest.

## Footnotes

^1^https://imagej.nih.gov/ij/
