# Supplementary material for: Hypomyelination and Oligodendroglial Alterations in a Mouse Model of Autism Spectrum Disorder
Source: Front Cell Neurosci. 2019 Jan 11;12:517. doi: 10.3389/fncel.2018.00517 (PMC6338056; doi:10.3389/fncel.2018.00517)

Supplementary figure 1

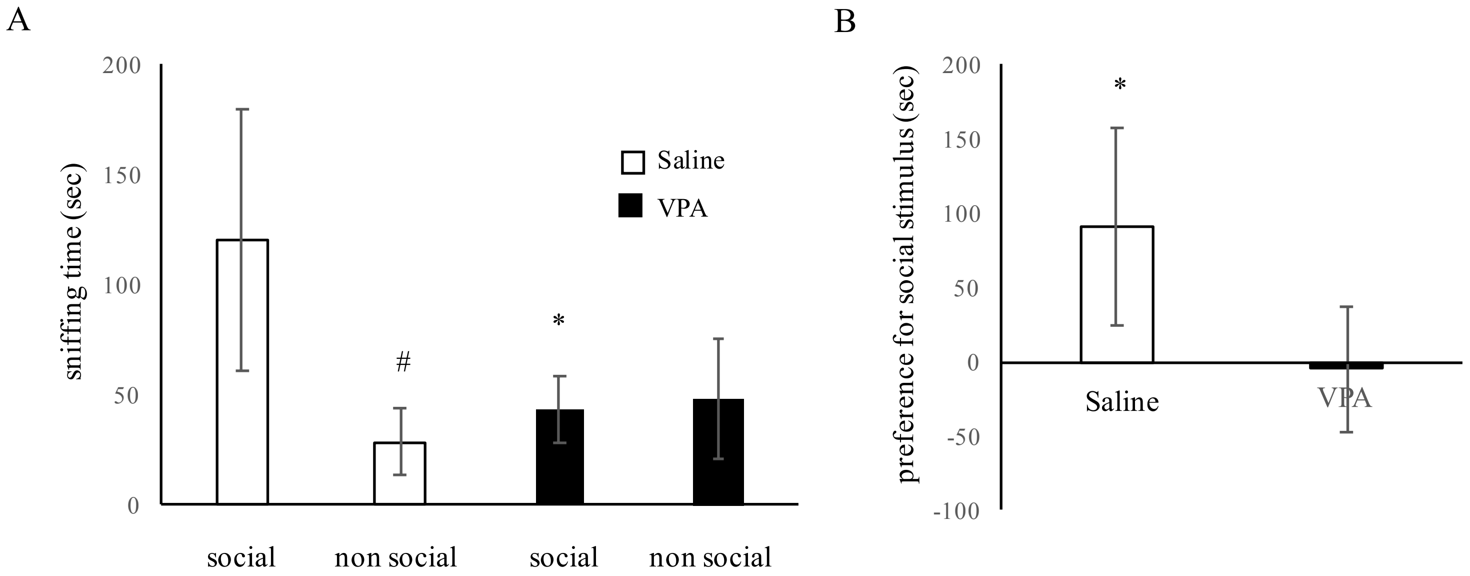

Supplementary figure 2

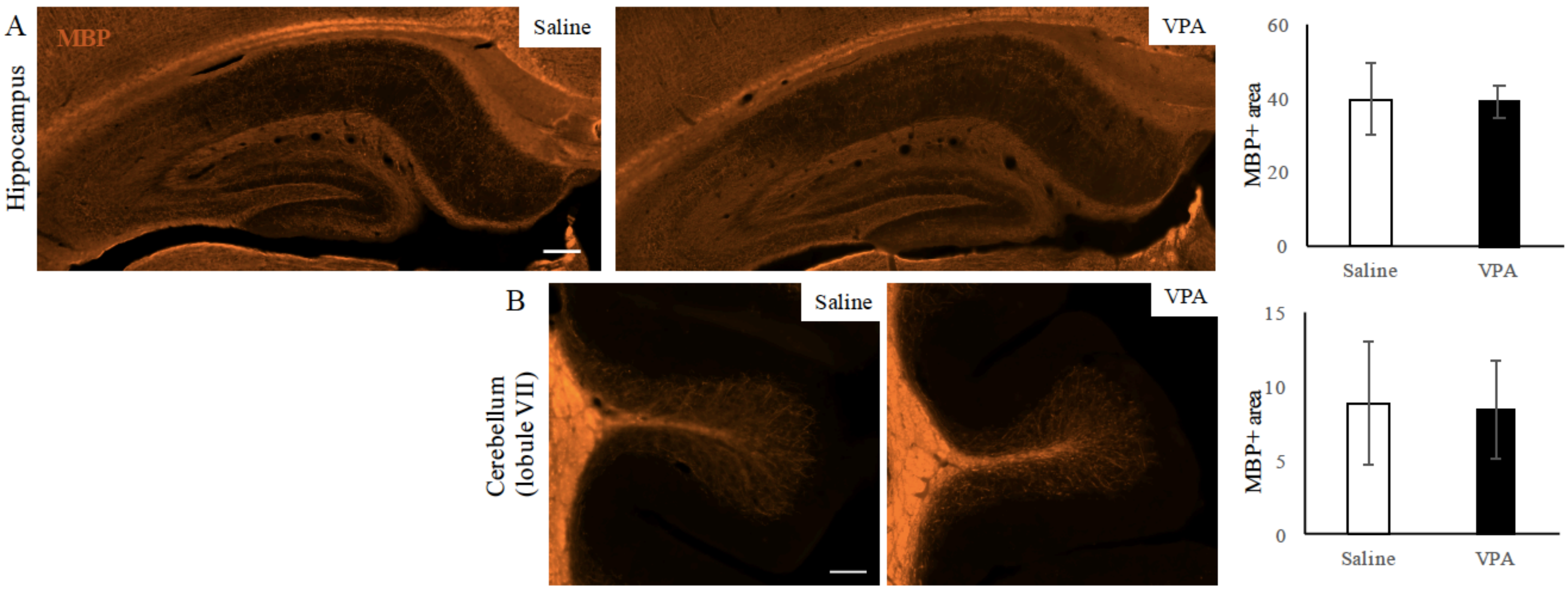

Supplementary figure 3

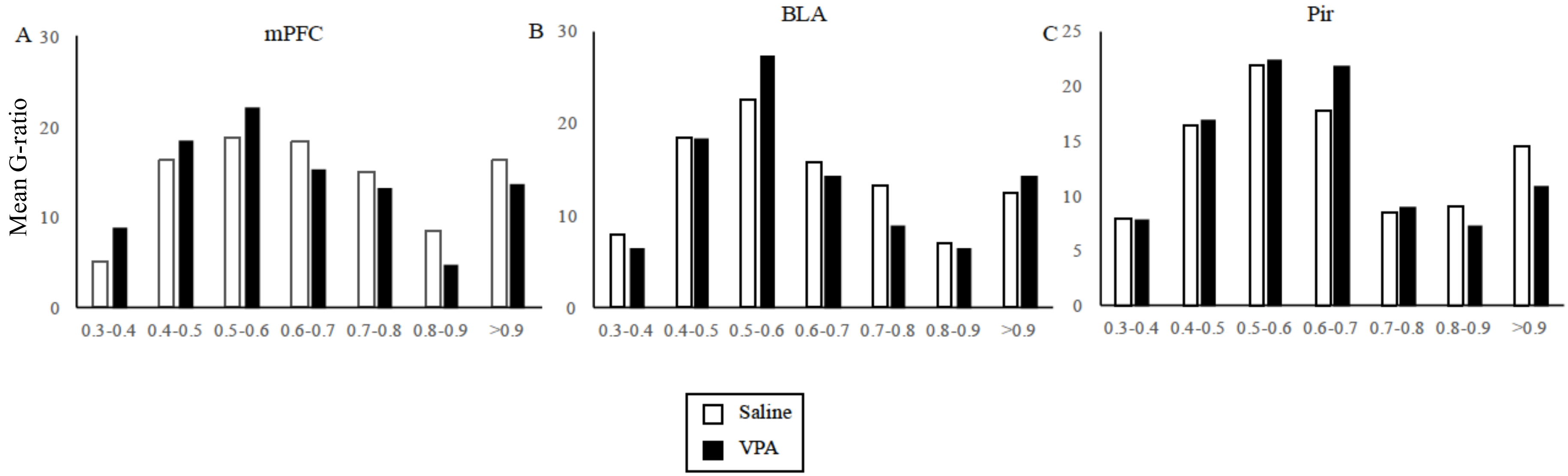

Supplementary figure 4

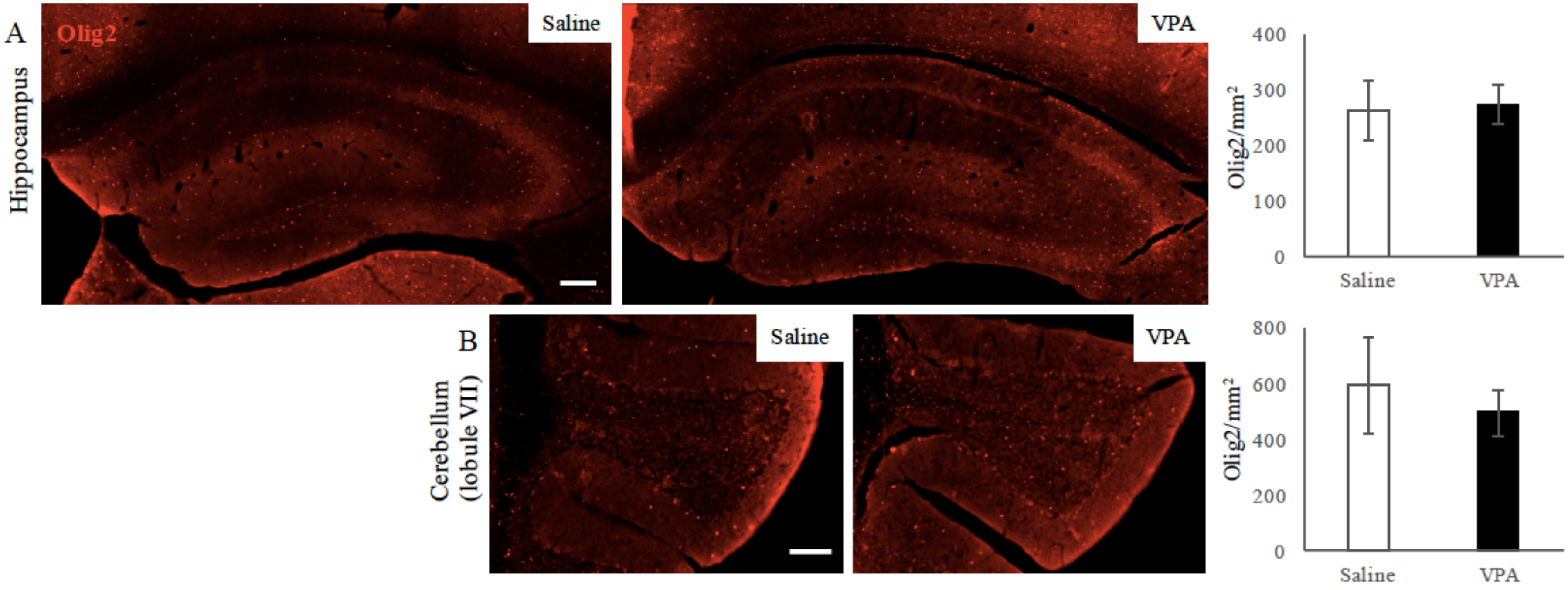

Supplementary figure 5

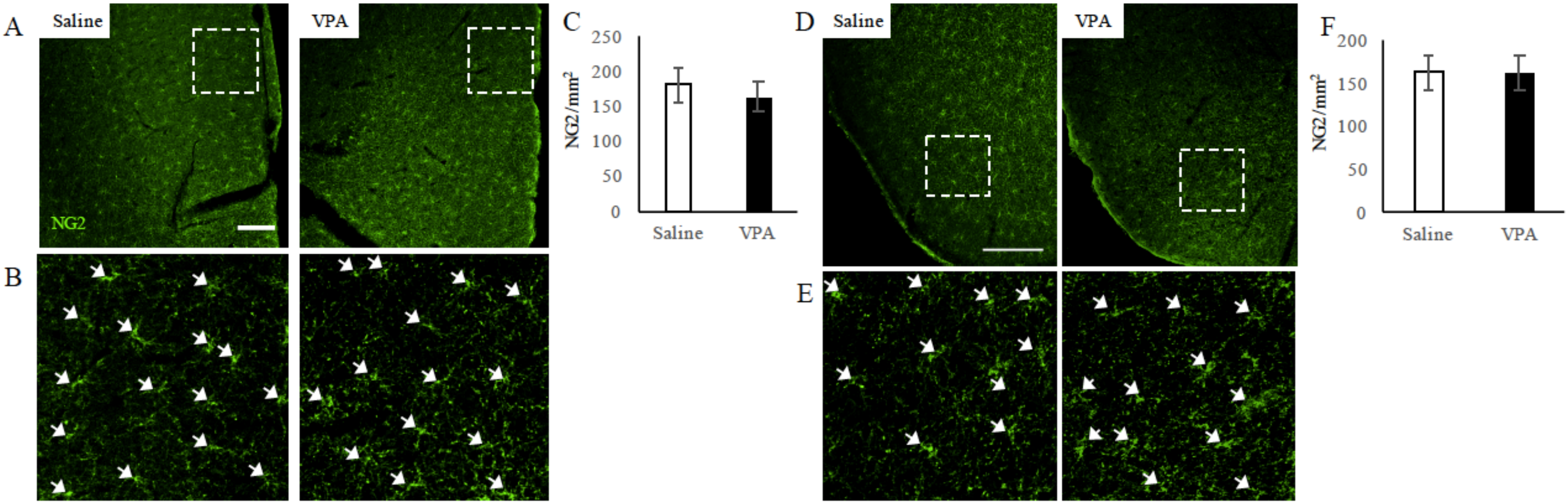

Supplement: Supplementary file 1 [file Data_Sheet_1.pdf]
