# Supplementary material for: Hypomyelination and Oligodendroglial Alterations in a Mouse Model of Autism Spectrum Disorder
Source: Front Cell Neurosci. 2019 Jan 11;12:517. doi: 10.3389/fncel.2018.00517 (PMC6338056; doi:10.3389/fncel.2018.00517)
Supplement: Supplementary file 2 [file Data_Sheet_2.docx]

SUPLEMENTARY FIGURE LEGENDS

**Supplementary figure 1. Animals prenatally exposed to VPA show reduced sociability.** (A) Saline-treated control animals spend more time sniffing the social side than the non-social side in the social interaction test, whereas VPA-treated animals show no differences in sniffing time. In addition, control animals spend more time sniffing the stimulus mouse (social side) than VPA-exposed animals. Saline/social side vs. VPA/social side: unpaired Student’s t test, *p < 0.05 Saline/social side vs. Saline/non-social side: paired Student t test, #p < 0.05. (B) Control animals show a preference for the social stimulus that is not observed in VPA-exposed animals. Unpaired Student’s t test, * p < 0.05. N = 5 mice/treatment. Data is presented as mean ± SD.

**Supplementary figure 2. Myelin content is not altered in the hippocampus and cerebellum upon VPA treatment.** MBP immunostaining in saline- and VPA-treated adult mouse brains (representative images, left panels) and estimated percentage of positive surface (right panels) in the following areas: A. Hippocampus, mean: saline: 39.89 ± 9.37; VPA: 39.04 ± 4.10; Student’s t-test: t=0.143, p=0.446 B. Cerebellum (lobule VII); mean: saline: 8.93 ± 5.18; VPA: 8.49 ± 3.28; Student’s t-test: t=0.126, p=0.452. Scale bars: 100 μm. N = 4-5 mice/treatment.

**Supplementary figure 3. Axonal diameter distribution shows no changes between saline- and VPA-treated animals.** Plots of axonal diameter ranges at the mPFC (**A**), BLA (**B**), and Pir (**C**).

**Supplementary figure 4. VPA treatment does not change OL-lineage cell numbers in the hippocampus and cerebellum.** Olig2 immunostaining in saline- and VPA-treated adult mouse brains (representative images, left panels) and Olig2-positive cell quantifications (right panels) in the following areas **A.** Hippocampus, mean: saline: 262.70 ± 51.79; VPA: 272.22 ± 34.29; Student’s t-test: t=-0.741, p=0.239. **B.** Cerebellum (lobule VII), mean: saline: 594.54 ± 174.38; VPA: 498.39 ± 82.22; Student’s t-test: t=1.006, p=0.174. Scale bars: 100 μm. N = 4-5 mice/treatment.

**Supplementary figure 5. VPA treatment does not alter OPCs in the mPFC and Pir.** NG2 immunostaining in saline- and VPA-treated adult mouse brains (representative images, left panels. Insets: lower left panels) and NG2-positive cell quantifications (right panels) in the following areas **A-C.** mPFC, mean: saline: 181.01 ± 26.06; VPA: 163.37 ± 20.29; Student’s t-test: t=0.965, p=0.189 **D-F.** Pir, mean: saline: 162.58 ± 20.35; VPA: 162.03 ± 20.38; Student’s t-test: t=0.037, p=0.486 Scale bars: 100 μm. Arrows: individual NG2 cells. N = 4-5 mice/treatment.
